# Methadone Potentiates the Cytotoxicity of Temozolomide by Impairing Calcium Homeostasis and Dysregulation of PARP in Glioblastoma Cells

**DOI:** 10.3390/cancers15143567

**Published:** 2023-07-11

**Authors:** Ondrej Honc, Jiri Novotny

**Affiliations:** Department of Physiology, Faculty of Science, Charles University, 128 00 Prague, Czech Republic

**Keywords:** glioblastoma, temozolomide, methadone, apoptosis, oxidative stress

## Abstract

**Simple Summary:**

Temozolomide is a widely used chemotherapeutic agent for the treatment of glioblastoma multiforme, but its efficacy is often severely limited by cell resistance to this treatment. Opioids have been found to increase the sensitivity of glioblastoma cells to temozolomide. However, the molecular mechanism underlying chemotherapeutic sensitization to temozolomide by methadone is unknown. Here, we observed that treatment of rat C6 glioblastoma cells and human U251 and T98G glioblastoma cells with a combination of these two drugs significantly reduced cell viability and increased apoptosis compared with temozolomide treatment alone. We found that the major factors involved in the observed effects include increased intracellular Ca^2+^ concentration, oxidative stress, PARP-1 protein cleavage, and impaired DNA integrity. The present study provides evidence for the potential value of methadone in the treatment of glioblastoma with temozolomide and sheds light on the molecular mechanism of synergistic action of these two drugs.

**Abstract:**

Methadone is commonly used as an alternative to morphine in patients with pain associated with glioblastoma and other cancers. Although concomitant administration of methadone and cytostatics is relatively common, the effect of methadone on the efficacy of cytostatic drugs has not been well studied until recently. Moreover, the mechanism behind the effect of methadone on temozolomide efficacy has not been investigated in previous studies, or this effect has been automatically attributed to opioid receptors. Our findings indicate that methadone potentiates the effect of temozolomide on rat C6 glioblastoma cells and on human U251 and T98G glioblastoma cells and increases cell mortality by approximately 50% via a mechanism of action independent of opioid receptors. Our data suggest that methadone acts by affecting mitochondrial potential, the level of oxidative stress, intracellular Ca^2+^ concentration and possibly intracellular ATP levels. Significant effects were also observed on DNA integrity and on cleavage and expression of the DNA repair protein PARP-1. None of these effects were attributed to the activation of opioid receptors and Toll-like receptor 4. Our results provide an alternative perspective on the mechanism of action of methadone in combination with temozolomide and a potential strategy for the treatment of glioblastoma cell resistance to temozolomide.

## 1. Introduction

Gliomas account for 80% of all CNS cancers and glioblastoma multiforme is one of the most severe forms [1]. The prognosis of glioblastoma multiforme (GBM), one of the most common types of gliomas, is very often unfavorable and depends on a number of factors. The most important factors are the operability of the tumor, the age of the patient and the sensitivity of the tumor to chemotherapy [2]. Due to its low molecular weight, associated with relatively good permeability through the hematoencephalic barrier, temozolomide (TMZ) is one of the drugs of first choice in the treatment of GBM. Resistance to TMZ, which is present in approximately 50% of GBM, significantly worsens the patient’s prognosis [3]. Because the main mechanism of TMZ action is DNA methylation, glioblastoma resistance to TMZ is primarily due to overexpression of O6-methylguanine-DNA methyltransferase [4], but other molecular mechanisms can also be involved [5].

The opioid receptor agonist methadone (MTD) is widely used in various therapies, including the treatment of opioid addiction [6], as a long-term analgesic for neuropathic pain syndromes, and in cancer therapy [7]. Comedication of MTD with cytostatics is therefore relatively common. The effect of MTD on the chemotherapeutic efficacy of some cytostatic drugs has recently been studied, but with conflicting results [8,9,10]. Nevertheless, few studies published to date have examined the mechanism of action of MTD on TMZ efficacy in more detail. Generally, it is automatically assumed that this action occurs through opioid receptors (OR) and TLR4 receptor [9]. Some recent works have explored the non-opioid mechanism of action of MTD in combination with chemotherapeutic agents [11,12]. The effect of MTD as a DNA-damaging agent and its effect on the expression of O6-methylguanine-DNA methyltransferase, one of the key mediators of TMZ resistance, was investigated as a possible mechanism to increase the susceptibility of cells to TMZ, but with negative results [12]. The effect of MTD on chemotherapeutic efficacy through caspase activation has also been described, but it was attributed to OR activation [13].

Still, in addition to the above characteristics, MTD, according to the current state of knowledge, has a broader pharmacological profile. From the point of view of pain therapy, its action as an NMDA receptor antagonist is important [14]. Some studies have also described other mechanisms of MTD actions, especially its effect on intracellular ROS production, intracellular calcium concentration and modulation of the respiratory chain in mitochondria [15]. These three effects may be closely related, and although they together may be responsible for a significant proportion of MTD-related side effects [16], as well as for the potential antitumor effect of MTD [17], so far very little attention has been paid to these effects. The aim of the present study was to investigate the ability of methadone to influence the efficacy of TMZ on model cell lines derived from GBM and, above all, to learn more about the mechanisms behind these effects. A more detailed description of these mechanisms could help to find an effective strategy to sensitize cells to treatment and possibly overcome the resistance of some GBMs to TMZ.

## 2. Materials and Methods

### 2.1. Cell Culture

Cell lines C6 (rat glioblastoma) and T98G and U251 (human glioblastoma) cell lines were purchased from the American Tissue Type Collection (Rockville, MD, USA). Cells were cultured in cell culture flasks to 80% confluence in a CO_2_ incubator under standard conditions (37 °C, 5% CO_2_ and humidified atmosphere). The cell culture medium (Dulbecco’s modified Eagle medium—high glucose) was supplemented with 10% fetal bovine serum (FBS), and antibiotic/antimycotic solution. Medium and antibiotics were purchased from Sigma Aldrich, and FBS was purchased from Gibco-Invitrogen (Carlsbad, CA, USA). For colorimetric assays, cells were seeded at a density of 10,000 cells per well in 96-well plates (100 μL medium in each well) and cultured in a CO_2_ incubator for 24 h, when they reached 70–80% confluence. For flow cytometry, cells were seeded at a density of 75,000 cells per well in a 24-well plate (1 mL medium in each well) or into 6-well plate at a density of 225,000 cells per well (in 3 mL of medium in each well). Cells attached to the surface and reached 70–80% confluence within the following 24 h. Before treating the cells, the cell medium was replaced with medium containing 1% FBS. The lower FBS concentration was used to slow cell proliferation and prevent cell overgrowth during the subsequent treatment, which lasted up to 72 h.

### 2.2. MTT Cell Viability Assay

For the MTT assay, after treatment of the cells in the 96-well plate, the cell medium was replaced with a solution of tetrazolium acid (Sigma-Aldrich, St. Louis, MO, USA) in medium containing 1% FBS and incubated for 30 min to allow the dye to be converted to insoluble purple formazan dye by the mitochondrial enzyme NADPH-dependent oxidoreductase. All but 25 μL of the medium was then aspirated from the wells, and the formazan was dissolved by adding 50 μL of DMSO (Sigma-Aldrich) to each well. Absorbance at a wavelength of 570 nm was measured using a Synergy HT spectrophotometer (BioTek Instruments, Winooski, VT, USA).

### 2.3. Western Blotting

Cells were harvested in ice-cold PBS (pH 7.4), centrifuged at 100× *g* (Hettich Universal 320 R, Andreas Hettich GmbH & Co. KG, Tuttlingen, Germany), and resuspended in TMES buffer containing cOmplete and PhosSTOP inhibitors (Roche, Basel, Switzerland). The cell suspension was then lysed by sonication with a Bandelin Sonopuls HD 2070 ultrasonic homogenizer (BANDELIN electronic GmbH & Co. KG, Berlin, Germany) (2 × 10 s, 50% intensity). Protein concentration was measured by the bicinchoninic acid (BCA) method using bovine serum albumin as a standard.

Samples for electrophoresis were prepared at a concentration of 1.5 μg/μL protein in Laemmli buffer, loaded at 22.5 μg per lane onto a 10% SDS-PAGE gel, and separated according to their size by applying 200 V for 45 min on an electrophoresis apparatus (Bio-Rad Laboratories, Hercules, CA, USA). Proteins were then transferred to nitrocellulose membrane (Amersham, Peapack, NJ, USA). Nonspecific binding sites were blocked with 5% dry milk dissolved in TBS-Tween buffer. The primary antibody against PARP-1 protein (9542S, Cell Signaling Technology; sc-8008, Santa Cruz Biotechnology, Inc., Dallas, TX, USA)) at a concentration of 1:5000 was used to detect selected proteins. Anti-mouse antibody NA931V and anti-rabbit antibody NA934V (GE Healthcare, Chicago, IL, USA) were used as secondary antibodies. Membranes were then incubated in chemiluminescent substrate (Life Technologies, Carlsbad, CA, USA) and developed on CP-BU X-ray film (Agfa, Motzel, Belgium). Ponceau staining was used to verify uniform loading and blots were quantified using ImageJ software (version 1.49).

### 2.4. Flow Cytometry

Cells were seeded at a density of 75,000 cells in a cultivation plate with 24 wells (1 mL medium in each well). Untreated cells were used as negative controls for cells exposed to MTD, naloxone, and TAK-242; cells treated with an equimolar concentration of DMSO were used as negative controls for cells exposed to TMZ. As a positive control for apoptosis, cells were treated with tert-butyl hydroperoxide (TBHP) at a final concentration of 200 μM for 2 h before flow cytometric analysis. Cells were incubated for 24, 48 and 72 h and then harvested by a 3-min trypsinization. Cells were then harvested in DMEM containing 10% FBS to inhibit trypsin activity and centrifuged 2 times for 3 min at 100× *g* at 4 °C in 96-well plates and washed with ice-cold PBS. After the second centrifugation, cells were resuspended in Annexin Binding Buffer (ABB; Apronex, Vestec, Czechia) and Annexin V conjugated to Dyomix 647 (Exbio, Vestec, Czechia) was added at a final concentration of 1 μL/100 μL of ABB. Cells were then incubated on ice in dark for 30 min. After incubation, the plate was centrifuged (3 min, 100× *g*, 4 °C) and resuspended in 150 μL of ABB. Ten minutes before measurement, Hoechst 33258 (Sigma Aldrich, St. Louis, MO, USA) was added to the cells at a final concentration of 1 μg/mL. Caspase 3 and 7 activation was assayed using the CellEvent^TM^ Caspase-3/7 Green Flow Cytometry Assay kit (Thermo Fisher Scientific, Waltham, MA, USA) according to the manufacturer’s instructions. All flow cytometric analyses were performed on LSRII flow cytometer (BD Biosciences, Franklin Lakes, NJ, USA) using the recommended optical configurations. Annexin V-Dyomix 647 was detected using the 640 ex 670/30 bp filter and Hoechst was measured using the 405 ex 450/40 bp filter. All flow cytometric data were analyzed using Kaluza Analysis 2.1 software (Beckman Coulter, Brea, CA, USA).

### 2.5. Determination of Oxidative Stress, Mitochondrial Potential, Mitochondrial Volume and Intracellular Ca^2+^ Concentration

Cells were seeded and treated in the same manner for 45 min as for Annexin V/Hoechst cytometry assay. Prior trypsinization, cells were incubated with 20 nM Mitotracker Red (Thermo Fisher Scientific, Waltham, MA, USA) for mitochondrial membrane potential (MMP) detection, 20 nM Mitotracker green (Thermo Fisher Scientific) for mitochondrial volume determination, 20 µM H2DCF-DA probe (Sigma Aldrich) for oxidative stress assessment, and 1 µM Fluo-4 AM (Thermo Fisher Scientific) for intracellular Ca^2+^ determination. As positive control, cells were stained with 100 μM TBHP, 10 μM antimycin and FCCP (Sigma Aldrich). After incubation, cells were trypsinized, centrifugated (3 min, 100× *g*, 4 °C) and transferred to a 96-well plate and directly analyzed by flow cytometry using the 488 ex 525/50 bp filter for detecting H2DCF-DA, Mitotracker Green and Fluo-4 AM fluorescence and the 561 ex 586/15 bp filter for detecting Mitotracker Red fluorescence.

### 2.6. TUNEL Assay

Cells were seeded at a density of 225 000 cells in a 6-well plate (3 mL medium in each well) and treated with TMZ or MTD or combination of both these drugs for 24, 48 and 72 h. Untreated cells were used as a negative control, positive control was part of the kit. After treatment, cells were trypsinized and fixed in PBS with 1% PFA for 15 min at −20 °C and cell membranes were permeabilized by incubation in 70% ethanol for 12 h at −20 °C. DNA was labeled by DNA labeling solution for 12 h at room temperature (RT), followed by staining cells with Alexa Fluor 488 dye-labeled anti-BrdU antibody for 30 min at RT. Thirty min before analysis, propidium iodide was added to the samples. Samples were measured by flow cytometry using the 488 ex 525/50 bp filter for detecting DNA fragmentation.

### 2.7. Statistics

When only two data sets were compared, an unpaired Student’s *t*-test was used. When more than two sets of data were compared, statistical significance was determined by analysis of variance (ANOVA) with Tukey’s multiple comparisons test. Only differences with *p*  <  0.05 were considered statistically significant.

## 3. Results

### 3.1. Effect of MTD on TMZ Efficacy in C6 Glioblastoma Cells

**Figure 1:**
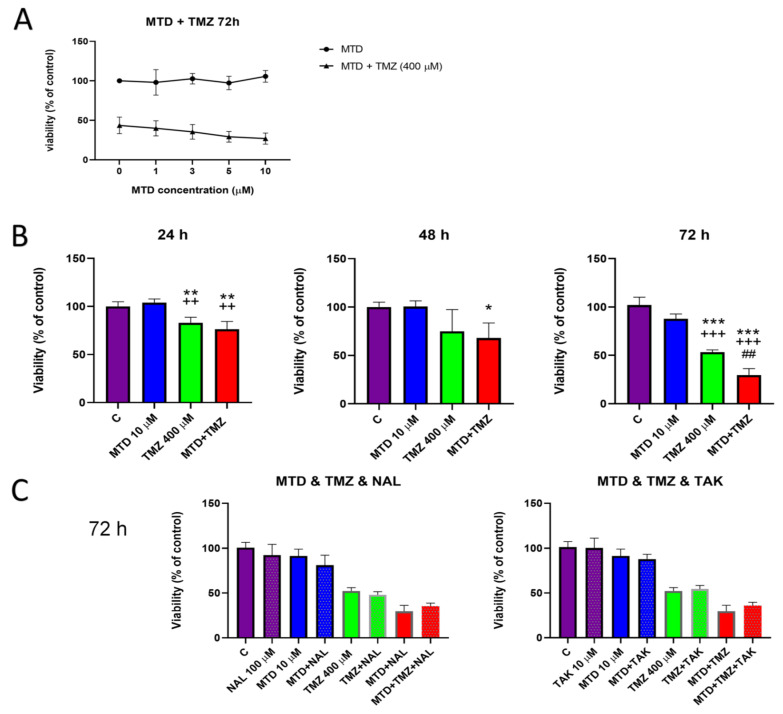
Effect of MTD on the efficacy of TMZ in C6 glioblastoma cells. Cells were treated for 24, 48 or 72 h. The MTT assay was performed after 72 h of treatment of cells for with increasing concentrations of MTD (1–10 µM) or after simultaneous treatment of cells with MTD and TMZ (400 μM), and the results were expressed as the percentage of cell viability compared to untreated controls (**A**). The MTT assay was performed after 24–72 h of treatment with MTD (10 µM), TMZ (400 µM) and a combination of these two drugs (**B**). MTT test was performed after concomitant treatment of cells with MTD (10 µM), naloxone (NAL; 100 µM) or TAK-242 (TAK; 10 µM) for 72 h (**C**). Each bar represents the mean ± SD of three independent experiments (*, *p* < 0.05 vs. control; **, *p* < 0.01 vs. control; ***, *p* < 0.001 vs. control; ^++^, *p* < 0.01 vs. MTD; ^+++^, *p* < 0.001 vs. MTD; ^##^, *p* < 0.01 vs. TMZ).

MTD at a concentration of 1 µM to 10 µM decreased the viability and proliferation of C6 glioblastoma cells treated with TMZ for 72 h in a concentration-dependent manner (Figure 1A). Cells treated with a combination of 10 µM MTD and 400 µM TMZ showed a decrease in viability by approximately 50% after 72 h (Figure 1B). Interestingly, the clear synergistic effect of combined treatment with MTD and TMZ was detectable only after 72 h. There was no significant difference between cells treated with TMZ together with MTD and those treated with TMZ alone after 24- and 48-h treatment (Figure 1B). Analogous results were obtained in experiments with both human glioblastoma cell lines treated for 72 h under the same conditions. A significant effect of the drug combination was observed in both cell lines compared with cells treated with TMZ alone (Supplementary Figure S1). Interestingly, MTD itself decreased cell viability of U251 cells by approximately 20% (Figure S1A), whereas no such effect was observed in T98G cells.

Pretreatment of cells with naloxone (100 µM) or TAK-242 (10 µM) for 1 h did not significantly alter the effect of 72 h of methadone treatment on TMZ efficacy (Figure 1C).

### 3.2. Effect of MDT and TMZ on Phosphatidylserine Localization in the Plasma Membrane

**Figure 2:**
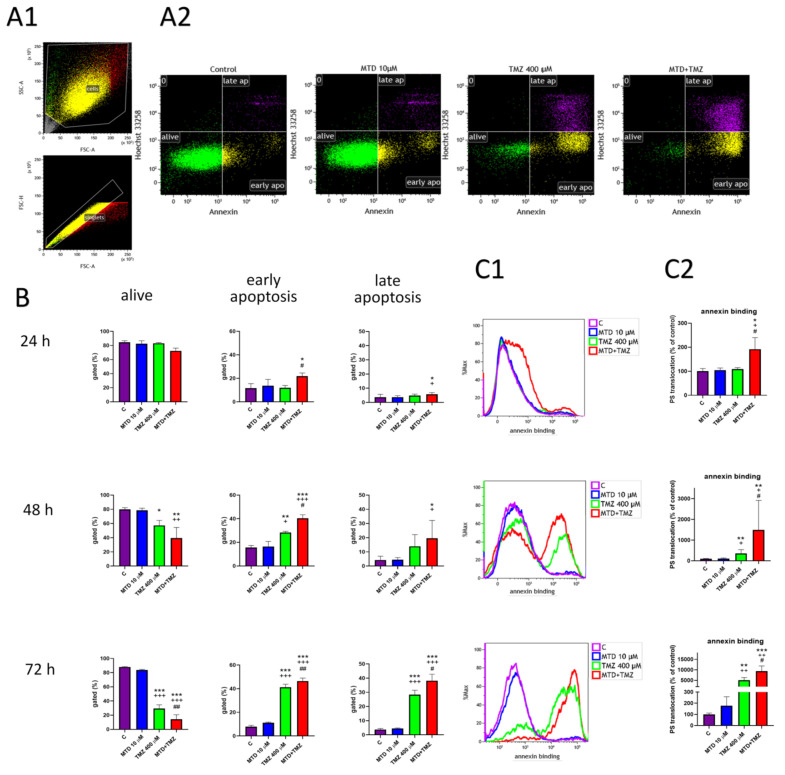
Effect of MTD, TMZ and a combination of these two drugs on the viability of C6 glioblastoma cells. Cells were treated with the drug tested (10 µM MTD, 400 µM TMZ) for 24, 48 or 72 h and then analyzed by flow cytometry using Annexin V and Hoechst 33258 staining to detect changes in apoptosis levels. Representative dot plots show the gating strategy (**A1**) and the effect of drugs after 72 h of treatment on the distribution of cells in quadrant gates (A—alive; E—early apoptosis; L—late apoptosis) (**A2**). Distribution of cells in gates (A, E and L) for each time interval of treatment (24, 48 and 72 h) (**B**). Representative overlay histograms of fluorescence intensity show the effect of MTD, TMZ, and a combination of these two drugs at different time points time points (24, 48 and 72 h) on the of binding Annexin V to phosphatidylserine (PS) (**C1**). Bar graphs show median fluorescence intensity corresponding to PS translocation (**C2**). Each bar represents the mean ± SD of three independent experiments (*, *p* < 0.05 vs. control; **, *p* < 0.01 vs. control; ***, *p* < 0.001 vs. control; ^+^, *p* < 0.05 vs. MTD; ^++^, *p* < 0.01 vs. MTD; ^+++^, *p* < 0.001 vs. MTD; ^#^, *p* < 0.05 vs. TMZ; ^##^, *p* < 0.01 vs. TMZ).

Using the Dynomix 647 conjugated Annexin V (A)/Hoechst 33258 (H) apoptosis kit for flow cytometry, we observed the time-dependent effect of treatment of C6 glioblastoma cells on the population distribution in the quadrants of the dot plots (Figure 2A). Translocation of phosphatidylserine (PS) (visualized by Annexin V staining) to the outer membrane is used as a marker for early and late apoptosis phase. As apoptosis progressed, a shift of cell population was observed from the “alive“ quadrant (not stained by A/H) to the “early apoptotis“ quadrant (stained by A only) and finally to the late apoptosis quadrant (stained by both A and H) (Figure 2(A2)).

After 24- and 48-h treatment, the effect of the combination of TMZ and MTD compared to TMZ alone was most evident in the shift of the cell population from the “alive” to the “early apoptosis“ quadrant (Figure 2B). This shift was driven by translocation of PS and was more visible when looking only at conjugated Annexin V fluorescence intensity (Figure 2(C1)); overlay graph of fluorescence intensity). The combination of MTD and TMZ increased the mean fluorescence in the Annexin V channel approximately 2-fold after 24 h of treatment and 5-fold after 48 h of treatment compared with cells treated with TMZ alone (Figure 2(C2)). After 72 h of treatment, the population of live cells decreased by approximately 50% (29% TMZ vs. 14% MTD+TMZ) (Figure 2B), consistent with the results shown about (Figure 1). In general, the translocation of PS to the outer cell membrane is one of the earliest markers of cell apoptosis, indicating possible ATP depletion [18]. The same effect was seen in U251 and T98G cells treated for 72 h (Supplementary Figure S2). The population of live cells decreased by approximately 50% in both cell lines (46% vs. 22% in U251 and 49% vs. 24% in T98G) when TMZ-treated cells were compared with the combination MTD+TMZ. Early and late apoptosis and translocation of PS, as measured by the Annexin binding assay, proceeded similarly after drug treatment in all cell lines tested.

### 3.3. Effect of MTD and TMZ on Mitochondrial Membrane Potential, Cytosolic Ca^2+^ Level and ROS Production

**Figure 3:**
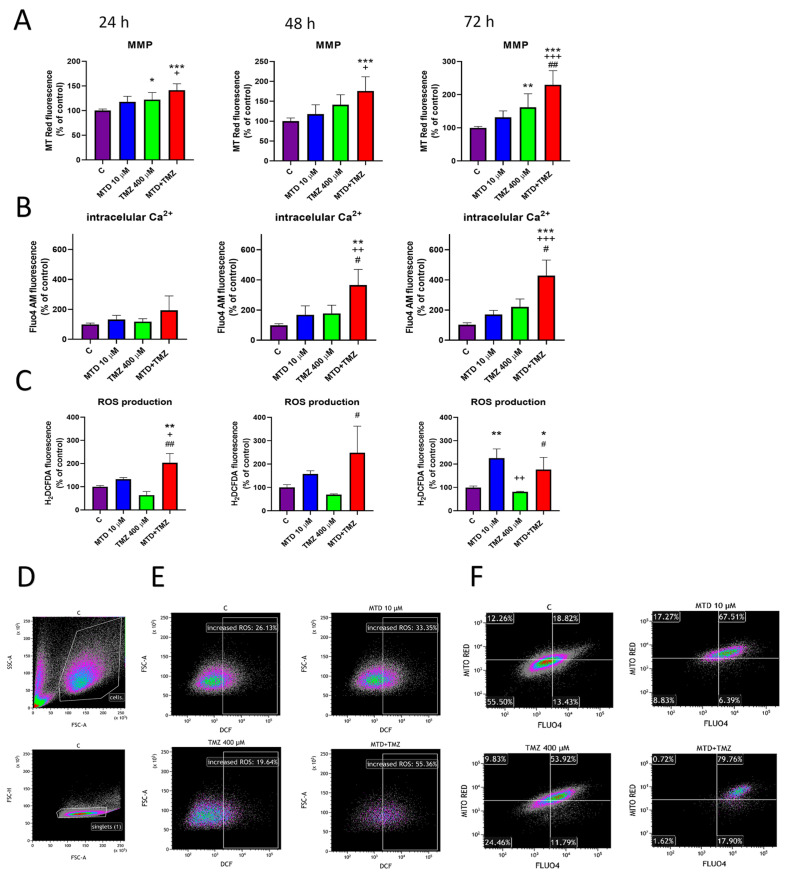
Effect of MTD, TMZ, and a combination of these two drugs on mitochondrial membrane potential, intracellular Ca^2+^ level, and ROS production. Cells were treated with the drug tested (10 µM MTD, 400 µM TMZ) for 24, 48 or 72 h and then analyzed by flow cytometry using MitoTracker Red (**A**), Fluo-4 AM probe (**B**), and H2DCF-DA (**C**). Representative dot plots show the gating strategy for determining H2DCF-DA fluorescence (**D**) and the effect of drugs after 72 h of treatment on the fluorescence signal intensity of H2DCF-DA in the selected gates (**E**), and on the cell distribution between quadrant gates as determined by the fluorescence intensity of Fluo-4 AM and Mitotracker Red probes (**F**). Each bar represents mean ± SD of three independent experiments (*, *p* < 0.05 vs. control; **, *p* < 0.01 vs. control; ***, *p* < 0.001 vs. control; ^+^, *p* < 0.05 vs. MTD; ^++^, *p* < 0.01 vs. MTD; ^+++^, *p* < 0.001 vs. MTD; ^#^, *p* < 0.05 vs. TMZ; ^##^, *p* < 0.01 vs. TMZ).

Using MitoTracker Red CMXRos and Fluo4 AM probes, we observed that MTD (10 µM; 24, 48, and 72 h) and TMZ (400 µM; 24, 48, and 72 h) alone slightly increased the level of mitochondrial membrane potential (MMP) (Figure 3A) and intracellular calcium concentration (Figure 3B) in C6 cells. Treatment of cells with a combination of MTD and TMZ showed a significant synergistic effect on MMP for all treatment time points (Figure 3A). Also, treatment with TMZ resulted in only a significant increase in MMP, whereas the effect of MTD was not significant, although we observed a slight increase at all treatment time points (Figure 3A). Treatment of cells with a combination of MTD and TMZ showed a significant enhancing effect on intracellular Ca^2+^ levels. Compared with untreated controls, there was a 2-, 3-, and 4-fold increase after 24, 48 and 72 h of treatment, respectively, whereas there was an approximately 2-fold increase at all measured time points of treatment with MTD+TMZ, compared with cells treated with TMZ alone (Figure 3B). Similar observations were made with the U251 and T98G cell lines. Although there was no statically significant difference between MMP in TMZ- and MTD+TMZ-treated cells, there was a marked difference in cytosolic Ca^2+^ levels in cells affected by either TMZ alone or by MTD+TMZ (Supplementary Figure S3). The increase in intracellular Ca^2+^ level measured with the Fluo-4 AM probe as a result of 72 h of treatment with MTD, TMZ and MTD+TMZ compared with untreated C6 glioblastoma cells is shown in Supplementary Figure S4.

The increase in mitochondrial potential was not accompanied by an increase in mitochondrial volume, as determined by the MitoTracker Green fluorescent probe. However, when C6 glioblastoma cells were treated with TMZ and a combination of TMZ and MTD for 72 h, small population of cells with increased MitoTracker Green fluorescence signal appeared, and this increase was accompanied by a decrease in mitochondrial potential, as measured by MitoTracker Red (Supplementary Figure S5; gate MG high). This population displayed a decreased signal in the FSC parameter (proportional to cell size) when backgated on the SSC/FSC dot plot (Supplementary Figure S5; represented as rare events by the blue color), which is typical of severely damaged cells in the advanced process of apoptosis. Significantly decreased mitochondrial potential indicates that the mitochondrial function of the cells is impaired, which is usually indicated by an increased Mitotracker Green signal [19].

Using the probe H2DCF-DA, we detected significantly increased level of ROS in MTD and MTD+TMZ treated cells compared to untreated controls (Figure 3C). After 24 h of treatment, the effect of the combination MTD and TMZ on the production of ROS was significantly stronger than in cells treated with MTD alone. In 48- and 72-h treated cells, MTD significantly increased the production of ROS compared with untreated controls. Interestingly, TMZ itself did not increase the production of ROS; on the contrary, the fluorescence signal was slightly and insignificantly decreased. The dot plots showing the gating strategy are displayed in Figure 3D. Representative dot plot diagrams (Figure 3E) demonstrate an increase in H2DCF-DA signal in MTD a MTD+TMZ treated cells (72 h), regardless of the cell size. A combination of Mitotracker Red and Fluo4 AM fluorescence revealed a synergistic increase in both measured parameters in cells treated simultaneously with MTD and TMZ for 72 h (Figure 3F).

### 3.4. Effect of MTD and TMZ on PARP-1 Cleavage and DNA Fragmentation

**Figure 4:**
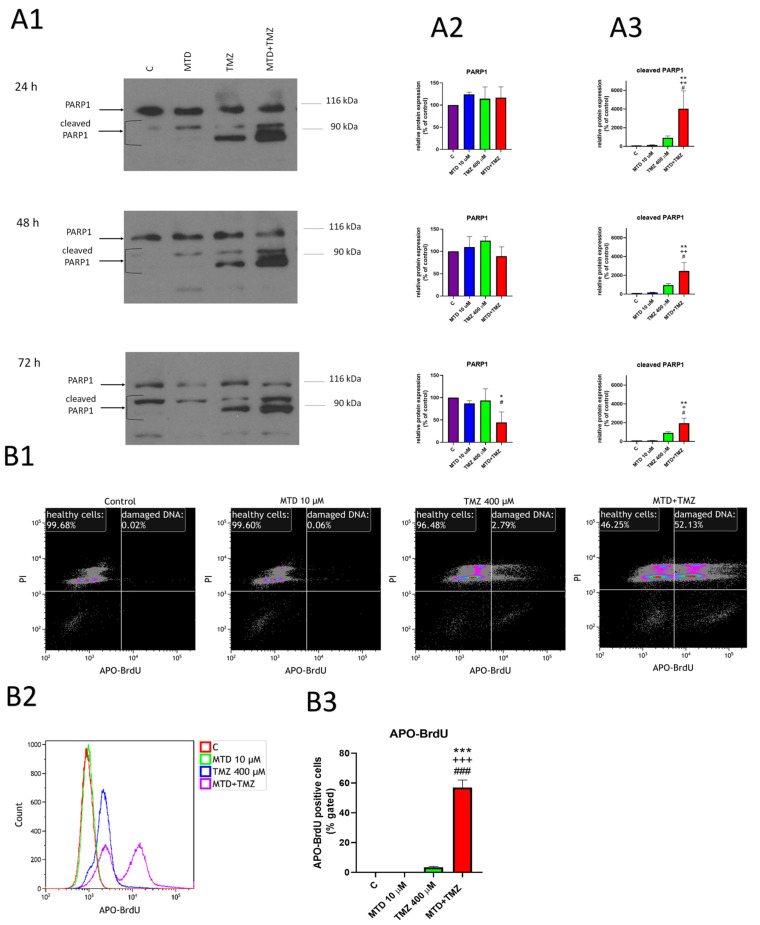
Effect of MTD, TMZ and a combination of these two drugs on PARP-1 protein expression and DNA integrity. After 24-, 48- or 72-h treatment of cells with the drug tested (10 µM MTD, 400 µM TMZ), the levels of PARP-1 and its cleaved forms were determined by Western blotting (**A**) and DNA integrity was determined by flow cytometry using a TUNEL kit (**B**). Bands on Western blots (**A1**) corresponding to the uncleaved and cleaved forms of PARP-1 were quantified (**A2**,**A3**) as described in Methods. Dot plots show the distribution of cell subsets as determined by TUNEL kit-based flow cytometric analysis of DNA integrity after 72 h of treatment (**B1**). An overlay histogram of the APO-BrdU signal (**B2**) and the percentage of APO-BrdU-positive cells (**B3**) after 72 h of treatment with the tested drugs. Each bar represents mean ± SD of three independent experiments (*, *p* < 0.05 vs. control; **, *p* < 0.01 vs. control; ***, *p* < 0.001 vs. control; ^+^, *p* < 0.05 vs. MTD; ^++^, *p* < 0.01 vs. MTD; ^+++^, *p* < 0.001 vs. MTD; ^#^, *p* < 0.05 vs. TMZ; ^###^, *p* < 0.001 vs. TMZ). The uncropped blots are shown in Figures S8 and S9.

The level of PARP-1 protein, which plays a crucial role in DNA repair but also in DNA degradation, was significantly altered after 24-, 48- and 72-h treatment of cells with TMZ or a combination of MTD and TMZ (Figure 4(A1)). As determined by Western blotting, the level of uncleaved PARP-1 protein (115 kDa) in cells treated with MTD+TMZ decreased after 72 h (Figure 4(A2)), whereas the level of cleaved PARP-1 protein (88 kDa) in cells treated with TMZ and MTD+TMZ increased significantly at all time intervals (Figure 4(A3)). Interestingly, the increase in cleaved PARP-1 protein at 24 and 48 h was not accompanied by a decrease in detected uncleaved PARP-1 protein. Increased PARP protein expression in response to some degree of DNA damage or other pathological conditions may play a role. Despite the large increase in cleaved PARP-1 protein, no increase in caspase activation was detectable with either drug combination or time interval of the treatment, as determined by flow cytometry using the caspase 3/7 activation kit (Supplementary Figure S6). A similar effect, a decrease in uncleaved PARP-1 protein, was observed in U251 cells treated with a combination MTD and TMZ for 72 h compared with control cells or cells treated with TMZ alone. Interestingly, this effect was not seen in T98G cells. Unfortunately, we were unable to detect the cleaved PARP-1 protein in human glioblastoma cells (Supplementary Figure S7). This may be due to the different species origin and specificity of the antibody used for the detection of PARP-1 in human and rat glioblastoma cell samples.

Next, we focused on DNA integrity as one of the possible key points in the mechanism of MTD-mediated effects. Using the Tunel assay and flow cytometry (Figure 4(B1)), we observed a significant increase in DNA fragmentation after 72 h of treatment of C6 glioblastoma cells with MTD+TMZ compared with TMZ treatment alone (57% vs. 3.34% APO-BrdU-positive cells). MTD itself did not increase DNA fragmentation, as shown by the median APO-BrdU signal (Figure 4(B2,B3)). This effect was not observed at 24- or 48-h treatment intervals.

## 4. Discussion

The effect of MTD on the efficacy of TMZ in the treatment of GBM has recently been studied with conflicting results [8,9]. We believe the main reason for these discrepancies is the different experimental designs, particularly the differences in MTD and serum concentrations and GBM models used. In particular, the serum concentration could play an important role because the sensitivity to drugs may vary under different cell culture conditions [20]. To date, few studies have looked more closely at the mechanism of action of MTD on TMZ efficacy in GMB treatment. The mechanism of MTD, which increases the efficacy of doxorubicine, a cytostatic used to treat other cancers, has been thought to be via blocking p-glycoprotein and thereby increasing drug concentration in the cells [10], but our current data suggest other mechanisms. Our data demonstrate that MTD at a final concentration of 10 µM significantly decreases the viability and proliferation of C6 glioblastoma cell when co-treated with TMZ (400 µM) for 72 h. The synergistic effect of co-treatment decreased the viability and proliferation of C6 glioblastoma cells by approximately 50%, compared to treatment with TMZ alone. At treatment times of 24 and 48 h, there were no visible effects on cell viability. However, we observed significant changes in other measured parameters, such as increase in MMP, PS translocation, and others. We also found that naloxone and TAK-242, antagonists of opioid and TLR4 receptors, respectively, did not significantly reduce the observed effects. Therefore, we aimed to further investigate the molecular mechanism behind the observed effects of MTD, which may help to elucidate some MTD adverse effects and outline possible strategies to improve the outcome of TMZ treatment.

The ability of MTD to affect the functional state of mitochondria and, in conjugation with this effect, to increase the intracellular amount of ROS and decrease ATP concentration, has been described previously [21]. Here, we observed that MTD at a concentration of 10 µM affected the functional state of mitochondria in terms of mitochondrial membrane potential (MMP). Mitochondrial volume remained unchanged under these conditions. Increased MMP could be due to impaired ATP synthesis or depletion of ADP as a substrate for ATP synthase [22]. Other pathological conditions may also be accompanied by increased ROS production [23].

We found that treatment of C6 cells with MTD resulted in increased intracellular calcium levels and ROS. The longer the treatment lasted, the more pronounced this effect was. The relationship between intracellular calcium homeostasis, ROS generation and mitochondrial activity is well documented, and disruption of homeostasis of any one of these factors can lead to disruption of the others, eventually resulting in opening of the mitochondrial permeability transition pore (mPTP) and cell death [24]. The functional state of mitochondria has far-reaching effects on a number of cellular processes, and its changes can lead to altered ATP production, changes increased ROS production, alterations of intracellular Ca^2+^ concentration, or release of apoptotic molecules, among others. At the same time, increased intracellular Ca^2+^ levels can result in increased ROS production, calcium influx into mitochondria and finally to mPTP opening, which is accompanied by MMP disruption and leads to cell death [24].

TMZ is an alkylating agent (delivering methyl groups to the purine bases of DNA) that causes DNA single and double strand breaks, ultimately leading to chromosome aberrations and apoptosis [25]. In addition to nuclear DNA, TMZ also interacts with mitochondrial DNA and causes detectable impairment of the mitochondrial respiratory chain, which is considered to be another mechanism of its action against cancer cells [26]. These two effects were observed in the present study. Resistance to TMZ, which is associated with a much worse overall prognosis of patients, is believed to be dependent mainly on the ability of cells to repair DNA damage. The DNA repair protein O6-methylguanine-DNA methyltransferase (MGMT) and its overexpression in GBM is one of the factors that determine TMZ resistance [27]. However, recent evidence shows that the crucial step for MGMT binding to DNA and its activation is the binding of PARP to MGMT and its PARylation [28]. Our results clearly show that TMZ leads to partial PARP-1 cleavage in C6 cells, and this effect is greatly enhanced by concomitant treatment with MTD. Another important (and possibly predominant) mechanism of TMZ resistance relates to the increase in mitochondrial fitness [29,30].

We have observed a strong effect of the combination of methadone and TMZ on several parameters. When cells were treated together with MTD and TMZ, the translocation of phosphatidylserine to the outer cell membrane, one of the indicators of early apoptosis, clearly outweighed the effects on overall cell viability. The changes, which were not reflected in the simple distribution of the population among quadrants, could be readily seen when comparing the overall fluorescence intensities. The distribution of phosphatidylserine (PS) mediated by ATP-dependent aminophospholipid translocase is tightly regulated and controlled in healthy cells and it serves as an “eat me“ [31]. However, recent evidence suggests mild translocation of PS can be reversed and need not always lead to cell death. Even a mild, sustained increase in intracellular ROS levels can lead to significant changes within cells, such as ATP depletion and eventual PS translocation to the outer layer of the cell membrane [31]. Importantly, increased intracellular Ca^2+^ can also regulate the activity of translocase [32].

Our observations showed that combined administration of MTD and TMZ to C6 cells significantly increased MMP at all time points, compared with untreated controls or cells treated with only one drug, indicating a significant effect on mitochondrial function. The ability of GBM to alter mitochondrial function in response to TMZ treatment has recently been described as one of the possible mechanisms of GBM resistance [29]. This is consistent with the observation that the sensitivity of GBM to TMZ was significantly increased by altering the functional state of mitochondria [33]. Thus, mitochondria appear to play one of the key roles in sensitivity to TMZ treatment and its overall efficacy. It is worth noting in this context that impaired mitochondrial function in brain progenitor/stem cells may reduce the rate of tumorigenesis [26]. Thus, the functional state and fitness of mitochondria are important factors determining the progress of tumorigenesis and sensitivity to TMZ treatment.

We found that simultaneous treatment of C6 cells with MTD and TMZ markedly increased intracellular Ca^2+^ concentration. Whereas MTD and TMZ alone caused approximately a 50–100% increase (depending on treatment duration) in fluorescence intensity of the Fluo4 AM calcium probe, the combination of the two drugs resulted in ~2-fold (24 or 48 h) and ~3-fold (72 h) increase in Fluo4-AM fluorescence signal compared to cells treated with the individual drugs. As mentioned above, intracellular calcium plays a critical role in many cellular processes, particularly those related to mitochondrial activity. Highly elevated intracellular Ca^2+^ levels promote increased influx of Ca^2+^ into mitochondria and cause the opening of mPTP, leading to the release of pro-apoptotic molecules, caspase activation and cell death [34]. Opening of mPTP can be accompanied by loss of MMP, which is evident in cells labeled for late apoptotic/death markers.

Increased intracellular Ca^2+^ levels induced by simultaneous treatment of cells with MTD and TMZ correlated well with the observed significant increase in oxidative stress. ROS production along with oxidative stress and increased intracellular Ca^2+^ levels are generally closely associated. Interestingly, we did not observe increased ROS levels in cells treated with TMZ alone, although TMZ is known to increase the production of ROS by interfering with mitochondrial DNA [35]. However, TMZ-resistant cell lines have been reported to prevent an increase in ROS after TMZ treatment through more permanent mitochondria coupling [27], suggesting that even TMZ-sensitive cell lines may have a mechanism to reduce the sensitivity of GBM to TMZ. The increased production of ROS, caused by treatment with MTD, may be the key to increasing TMZ efficacy. Increased ROS can also promote DNA single- and double-strand breaks and activation of PARP, eventually leading to ADP depletion and impaired ATP production [24]. Furthermore, we found a significant synergistic effect of the combination of MTD and TMZ on PARP-1 protein cleavage. PARP-1 acts as a DNA repair protein under standard conditions [36], and its activity may be related to tumor cell resistance to TMZ by mediating DNA break repair of [24] and possibly activating MGMT [28]. However, upon massive DNA damage or cleavage of PARP-1 during activation of apoptotic and necrotic pathways, its activity is altered, and its fragments may contribute significantly to DNA degradation [33]. Although activation of apoptotic caspase pathways is generally considered to be one of the major pathways leading to PARP-1 cleavage, there are also non-caspase mechanisms of PARP-1 cleavage that may be related to calcium dysregulation and increased intracellular ROS levels [37]. Interestingly, we found here that PARP-1 cleavage did not result in a significant decrease in uncleaved PARP-1 protein. Its level was highest at shorter treatment durations (24 h for cells treated with TMZ alone and 24 or 48 h for cells treated with TMZ+MTD), and that it increased after 72 h of treatment for cells treated with either TMZ alone or TMZ+MTD compared with untreated controls. We hypothesize that this is likely due to an increased number of DNA single- and double-strand breaks causing increased expression of PARP-1 protein and ultimately leads to replicative stress, which is consistent with the observed effects of some DNA-interfering cytostatic drugs [38] and also with our results after 72 h of treatment with TMZ or MTD+TMZ. Importantly, DNA breaks were detected with a much higher signal in the case of MTD+TMZ than TMZ alone. In this context, it may be important to point out that overexpression and hyperactivation of PARP-1 has been reported to ultimately lead to depletion of ADP (possibly contributing to the increase in MMP) and possibly apoptosis as a consequence of intracellular ATP depletion [38].

## 5. Conclusions

Our present study demonstrated that the enhancing effect of methadone on temozolomide efficacy in C6 glioblastoma cells is not mediated by opioid receptors or TLR receptors and that other mechanisms must be involved. Our results suggest that MTD affects the functional state of mitochondria. The other detected effects of MTD were closely related to mitochondrial state, such as changes in intracellular Ca^2+^ concentration, increased oxidative stress and changes intracellular ATP levels. These changes induced by MTD may contribute to the observed decreased DNA integrity and cleavage of PARP-1 protein and the translocation of phosphatidylserine to the outer cell membrane. Because PARP-1 plays an important role in maintaining DNA integrity and also in the depletion of ADP, alterations (either cleavage or increased expression) of this protein may have strong effects on cell survival. Therefore, our results suggest that modulation of mitochondrial functional state may increase the efficacy of TMZ and reduce the resistance of GBM to cytostatic drugs. Since MTD has a low profile of potential adverse effects and the combination of TMZ and MTD is widely used in the clinical treatment of cancer, further research is needed on the mechanism of action of MTD in TMZ-treated GBM. A better understanding of these mechanisms could help to find new strategies to overcome or at least attenuate the resistance of GBM to cytostatic treatment. The results on rat C6 glioblastoma cells were confirmed by a series of selected experiments with U251 and T98G glioblastoma cells of human origin. Both cell lines showed similar changes in all measured parameters such as cell proliferation and viability, intracellular Ca^2+^ concentration, MMP and PARP cleavage when treated by the same drug combinations for 72 h. Therefore, it can be assumed that the mechanisms behind the enhancing effect of MTD on TMZ-mediated cytotoxicity are similar in different glioblastoma cells.

## Data Availability

The data presented in this study are available in this article (and Supplementary Material). Further information is available from the corresponding author on reasonable request.

## Supplementary Materials

The following supporting information can be downloaded at https://www.mdpi.com/article/10.3390/cancers15143567/s1: Figure S1: Effect of MTD on the efficacy of TMZ in U251 (A) and T98G (B) glioblastoma cells. Figure S2: Effect of MTD, TMZ and a combination of these two drugs on the viability of U251 (A) and T98G (B) glioblastoma cells; Figure S3: Effect of MTD, TMZ, and a combination of these two drugs on mitochondrial membrane potential and cytosolic Ca^2+^ level in U251 and T98G glioblastoma cells; Figure S4: Effect of 72-h treatment with MTD and TMZ on mitochondrial membrane potential and mitochondrial volume of C6 glioblastoma cells; Figure S5: Effect of 72-h treatment with MTD, TMZ, and a combination of these two drugs on intracellular Ca^2+^ levels in C6 glioblastoma cells; Figure S6: Representative dot plots showing the effect of 72-h treatment of C6 glioblastoma cells with MTD, TMZ, and a combination of these two drugs on caspase 3 and 7; Figure S7: Effect of MTD, TMZ and a combination of these two drugs on PARP-1 protein expression in U251 and T98G glioblastoma cells; Figures S8 and S9: Examples of original images of immunoblots and Ponceau staining.
