# Supplementary material for: Methadone Potentiates the Cytotoxicity of Temozolomide by Impairing Calcium Homeostasis and Dysregulation of PARP in Glioblastoma Cells
Source: Cancers (Basel). 2023 Jul 11;15(14):3567. doi: 10.3390/cancers15143567 (PMC10377588; doi:10.3390/cancers15143567)
Supplement: Supplementary file 1 [file cancers-15-03567-s001.zip › cancers-2153439-supplementary.pdf]

Supplementary Materials

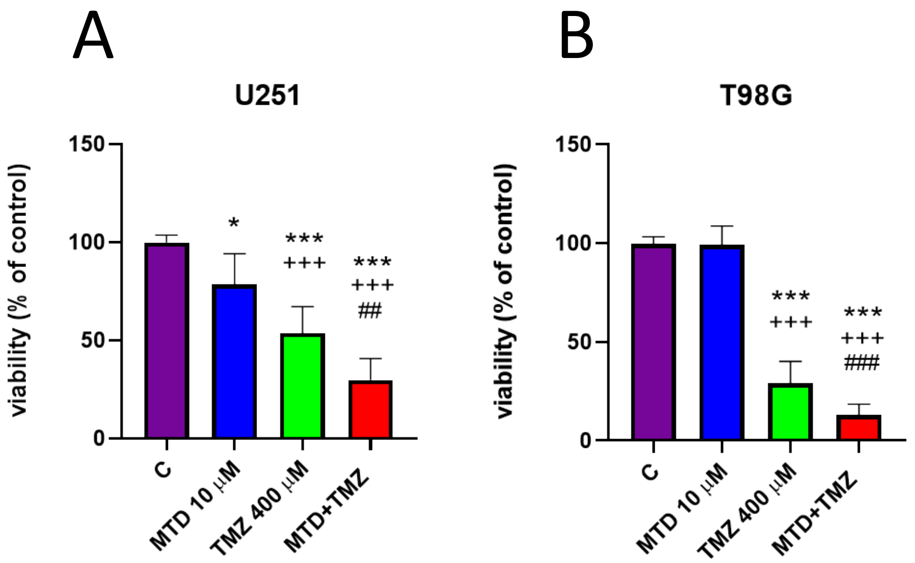

Supplementary Figure S1. Effect of MTD on the efficacy of TMZ in U251 (A) and T98G (B) glioblastoma cells. The MTT assay was performed after 72 hours of treatment with MTD (10 μM), TMZ (400 μM) and a combination of these two drugs. Each bar represents the mean ± SD of at least three independent experiments (*, $p < 0.05$ vs. control; ***, $p < 0.001$ *vs.* control; +++, $p < 0.001$ *vs.* MTD; ##, $p < 0.01$ *vs.* TMZ; ###, $p < 0.001$ *vs.* TMZ).

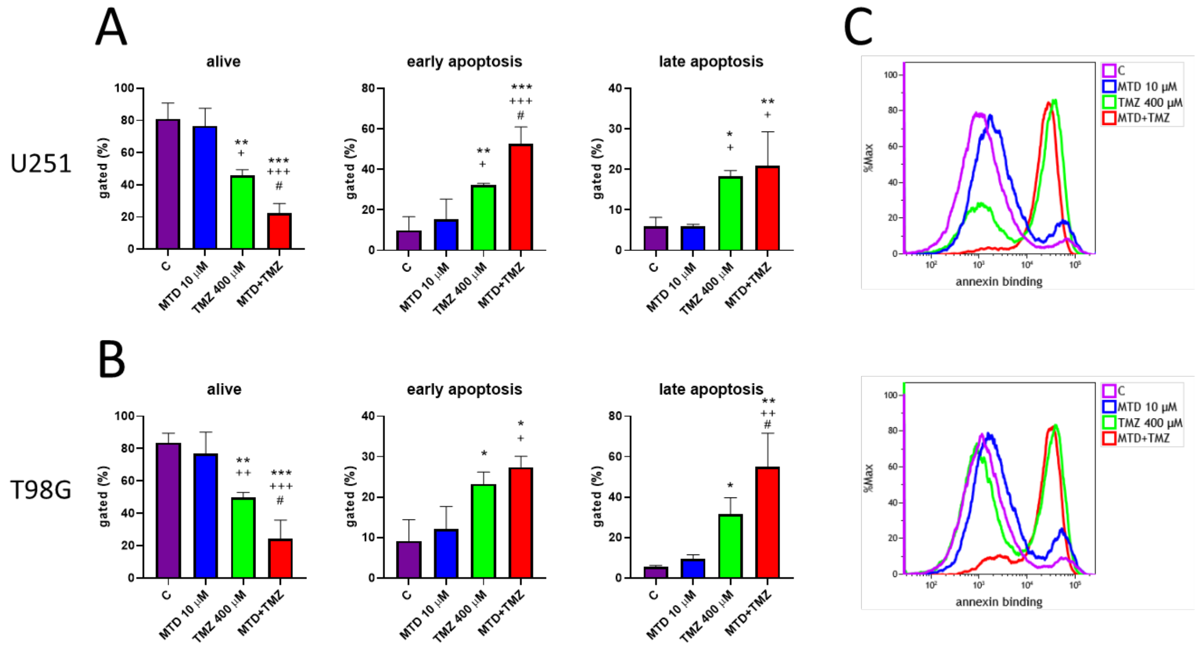

Supplementary Figure S2. Effect of MTD, TMZ and a combination of these two drugs on the viability of U251 (A) and T98G (B) glioblastoma cells. Cells were treated with the drug tested (10 µM MTD, 400 µM TMZ) for 72 hours and then analyzed by flow cytometry using Annexin V and Hoechst 33258 staining to detect changes in apoptosis levels. Bar graphs shows distribution of the cells within gates (alive, early apoptosis, late apoptosis). Same gating strategy as in Figure 2 (A1, A2) was used. Representative overlay histograms of fluorescence intensity show the effect of MTD, TMZ, and a combination of these two drugs at 72 hours treatment on the of binding Annexin V to phosphatidylserine (PS) after its translocation (C). Each bar represents the mean ± SD of three independent experiments (*, $p < 0.05$ *vs.* control; **, $p < 0.01$ *vs.* control; ***, $p < 0.001$ *vs.* control; $^{+}$, $p < 0.05$ *vs.* MTD; $^{++}$, $p < 0.01$ *vs.* MTD; $^{+++}$, $p < 0.001$ *vs.* MTD; $^{\#}$, $p < 0.05$ *vs.* TMZ).

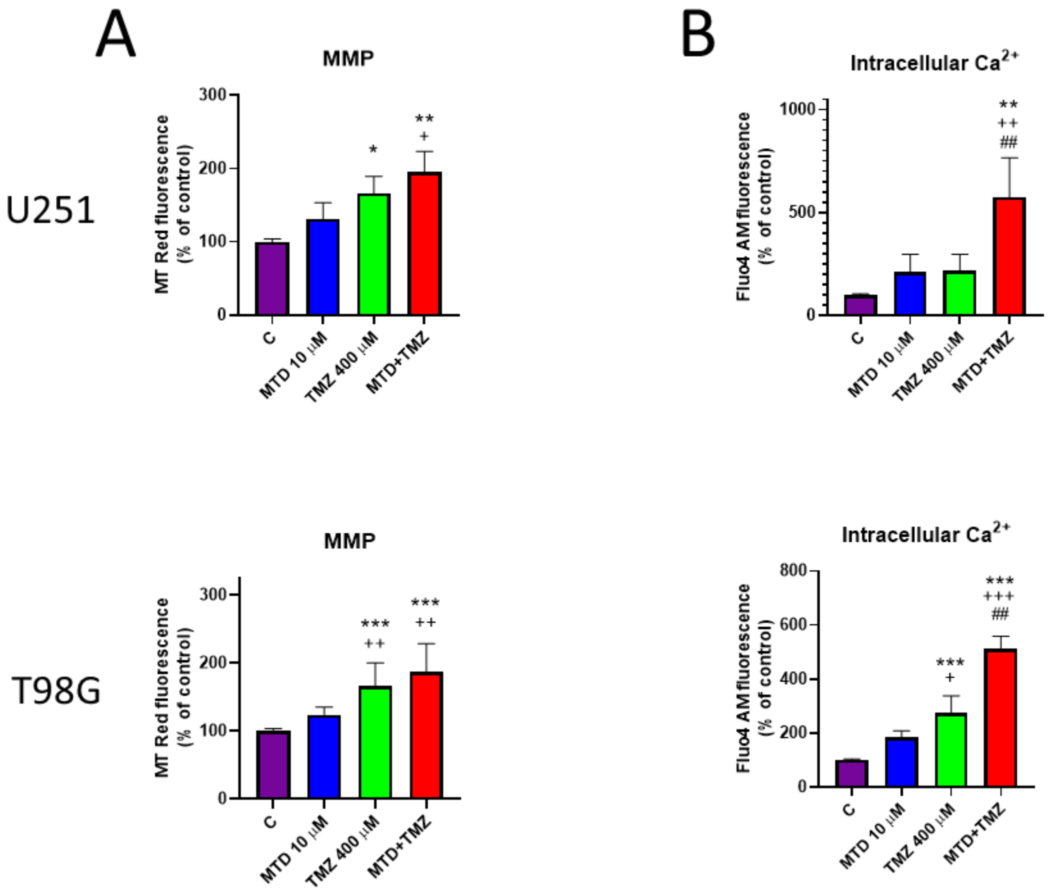

Supplementary Figure S3. Effect of MTD, TMZ, and a combination of these two drugs on mitochondrial membrane potential and cytosolic Ca^{2+} level in U251 and T98G glioblastoma cells. Cells were treated with the drug tested (10 μM MTD, 400 μM TMZ) for 72 hours and then analyzed by flow cytometry using MitoTracker Red (A) and Fluo-4 AM probe (B). Each bar represents mean ± SD of three independent experiments (*, $p < 0.05$ *vs.* control; **, $p < 0.01$ *vs.* control; ***, $p < 0.001$ *vs.* control; $^{+}$, $p < 0.05$ *vs.* MTD; $^{++}$, $p < 0.01$ *vs.* MTD; $^{+++}$, $p < 0.001$ *vs.* MTD; $^{\#\#}$, $p < 0.01$ *vs.* TMZ).

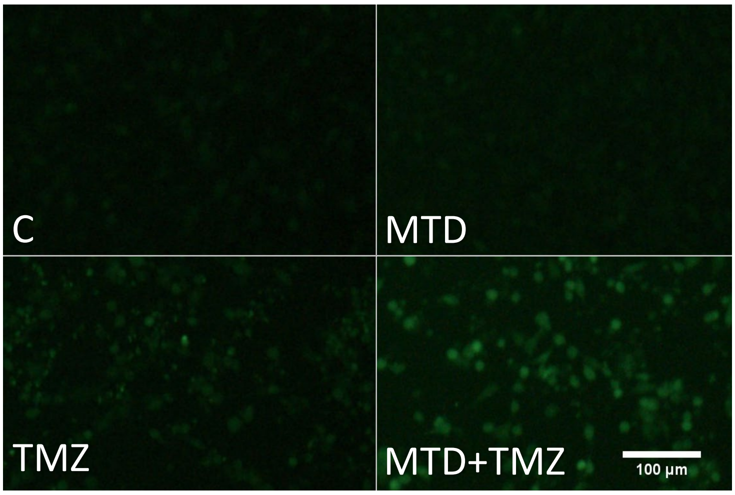

Supplementary Figure S4. Effect of 72-hour treatment with MTD, TMZ, and a combination of these two drugs on intracellular Ca^{2+} levels in C6 glioblastoma cells. Representative photomicrographs show the increase in Fluo-4 AM fluorescence signal reflecting the increased Ca^{2+} levels in cells treated with MTD (10 µM), TMZ (400 mM), or MTD+TMZ (M+T) for 72 hours.

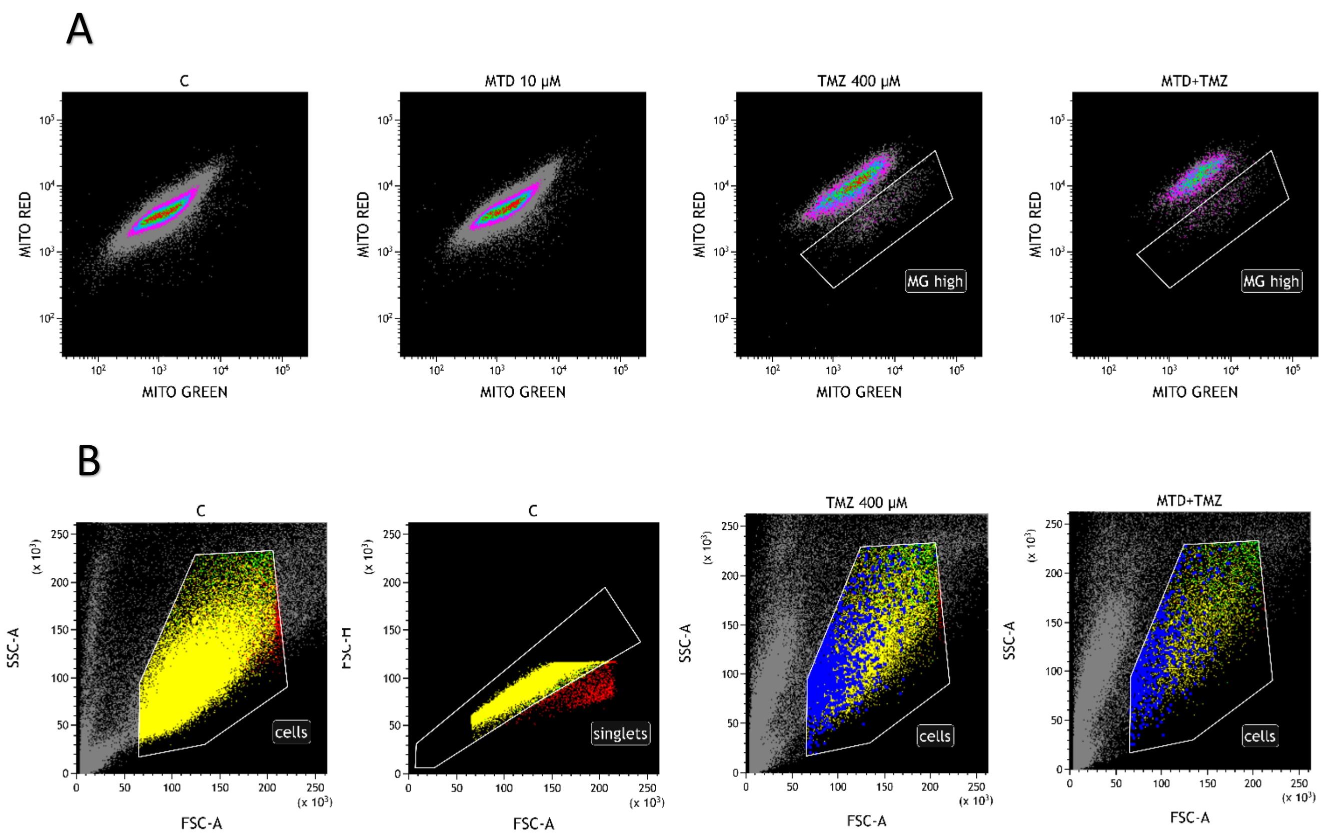

Supplementary Figure S5. Effect of 72-hour treatment with MTD (10 µM), TMZ (400 mM), and a combination of these two drugs (MTD+TMZ) on mitochondrial membrane potential and mitochondrial volume of C6 glioblastoma cells. Mitochondrial membrane potential and mitochondrial volume in control (C) and drug-treated cells were measured by flow cytometry using Mitotracker Red (MR) and Mitotracker Green (MG), respectively. Representative dot plots showing the effect of treatment on the fluorescence intensity of MR and MG in a subset of cells **(A).** Representative SSC/FSC dot plots showing the gating strategy and backgating of the “MG high“ gate **(B).** The blue color represents rare cell events from the “MG high“ gate.

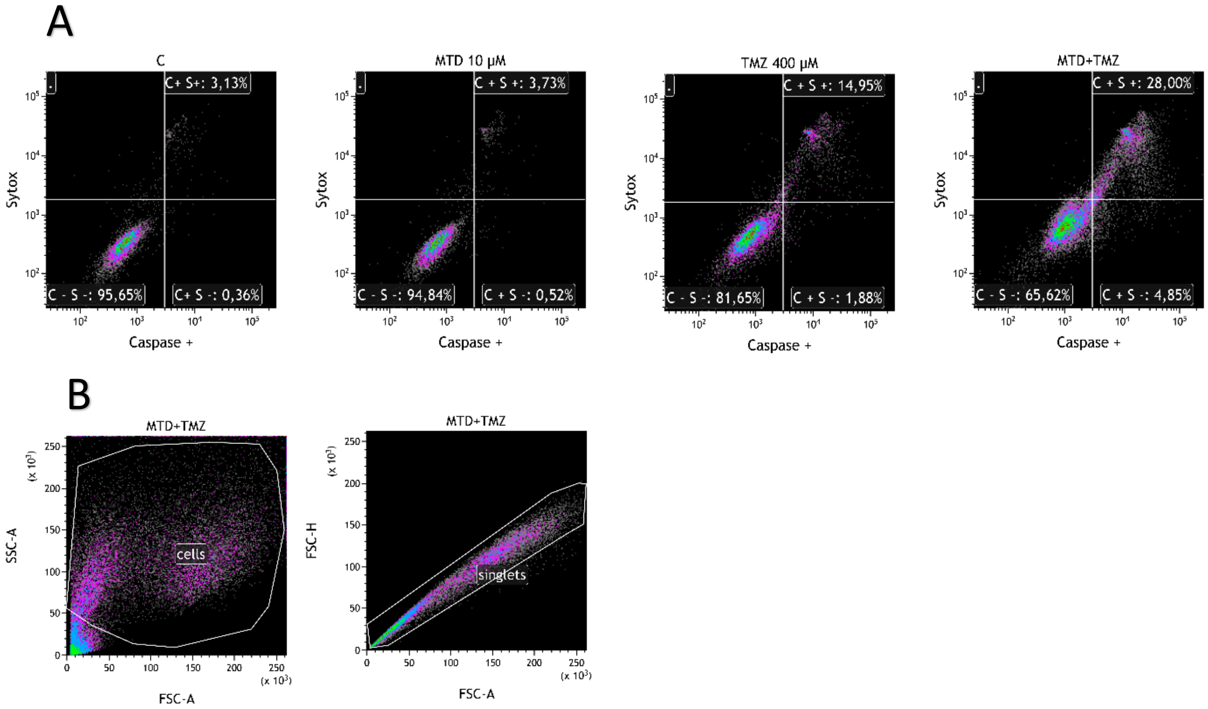

Supplementary Figure S6. Representative dotplots showing the effect of 72-hour treatment of C6 glioblastoma cells with MTD (10 µM), TMZ (400 mM), and a combination of these two drugs (MTD+TMZ) on activation of caspase 3 and 7, using the CellEvent^{TM} Caspase-3/7 Green Flow Cytometry Assay kit (ThermoFisher). Live cells without activation of caspases 3 and 7 (Casp 3/7) are negative for both Sytox staining (S) and CellEvent capase staining (C). Cells in the late phase of apoptosis and dead cells are stained with both C and S and are in the C+S+ gate. Cells with activated caspases 3 and 7 form a distinct population in the C+S- gate **(A)**. Representative SSC/FSC dot plots showing the gating strategy **(B)**.

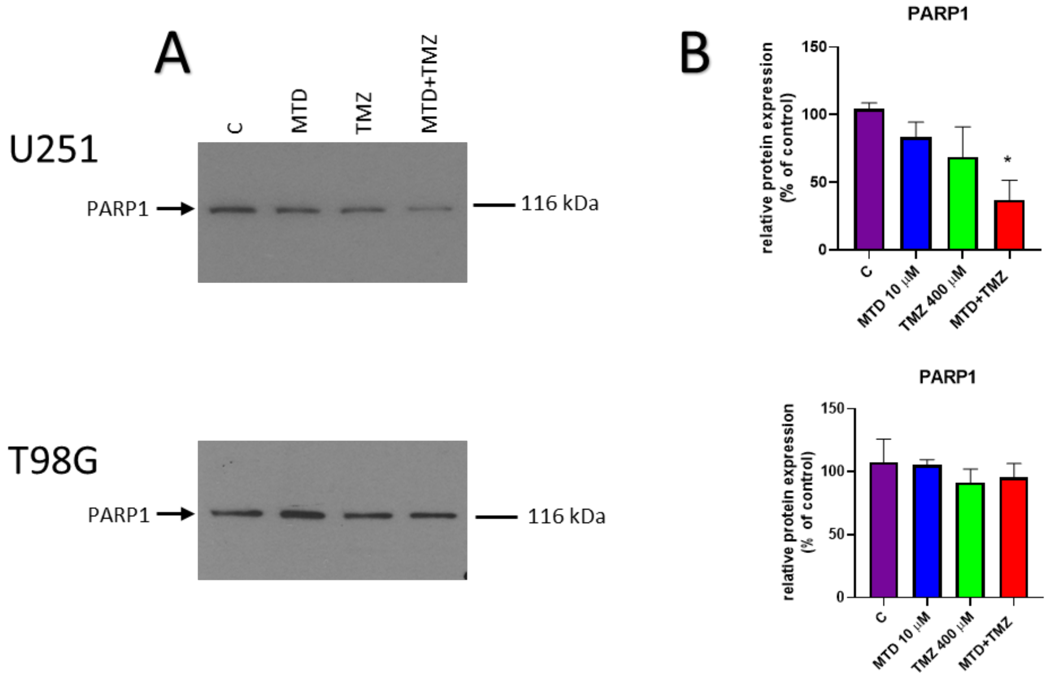

Supplementary Figure S7. Effect of MTD, TMZ and a combination of these two drugs on PARP-1 protein expression in U251 and T98G glioblastoma cells. After 72-hour treatment of cells with the drug tested (10 μM MTD, 400 μM TMZ), the levels of PARP-1 were determined by Western blotting. Bands on Western blots (A) corresponding to the uncleaved forms of PARP-1 were quantified (B) as described in Methods. Each bar represents mean ± SD of three independent experiments (*, $p < 0.05$ *vs.* control).

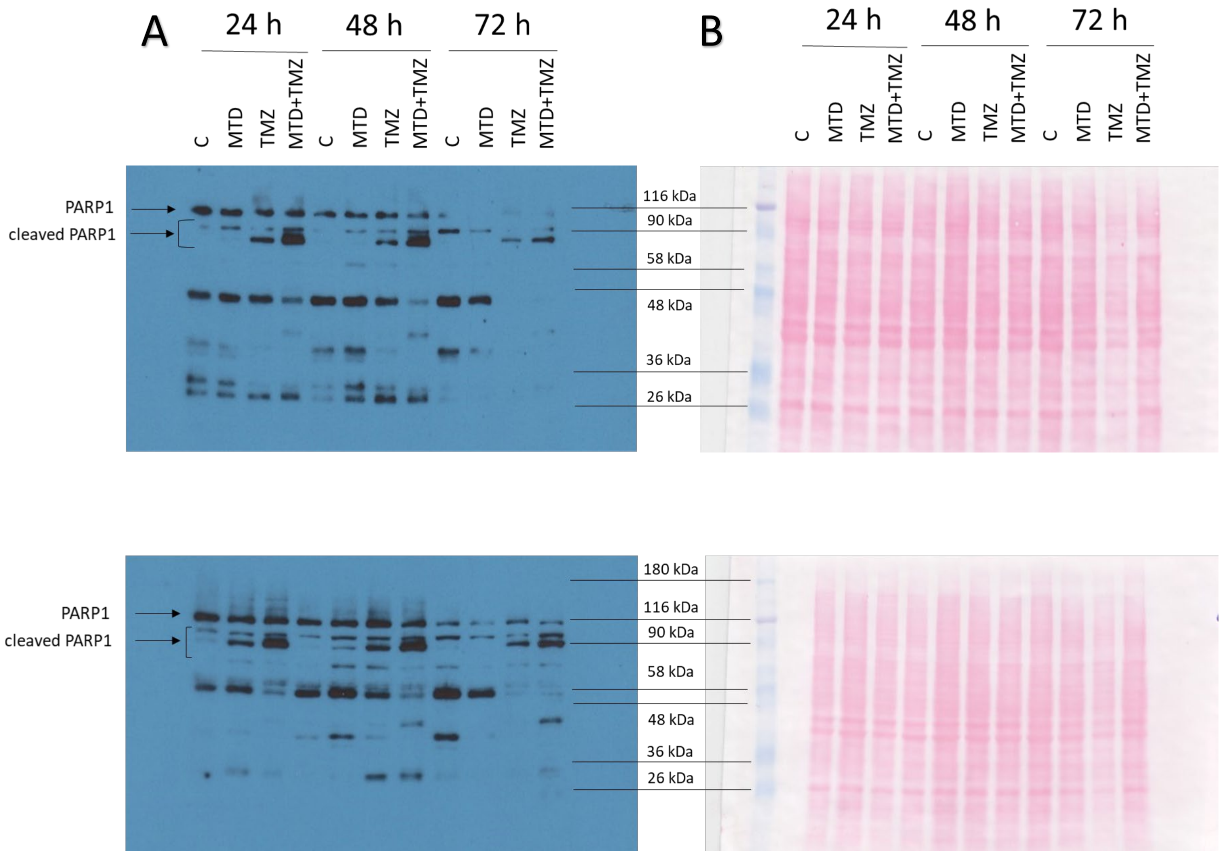

Supplementary Figure S8. Examples of original images of immunoblots and Ponceau staining. C6 glioblastma cells were treated with MTD (10 µM), TMZ (400 mM), or MTD+TMZ for different time intervals, and cell homogenates were subjected to SDS-PAGE and then, before immunoblotting with antibody to PARP1 protein, stained with Ponceau S to normalize loading. Shown are examples of Western blots of samples prepared from cells treated for 24, 48 and 72 hours with the tested drugs and their combinations (A) and images of the corresponding nitrocellulose membranes stained with Ponceau S (B).

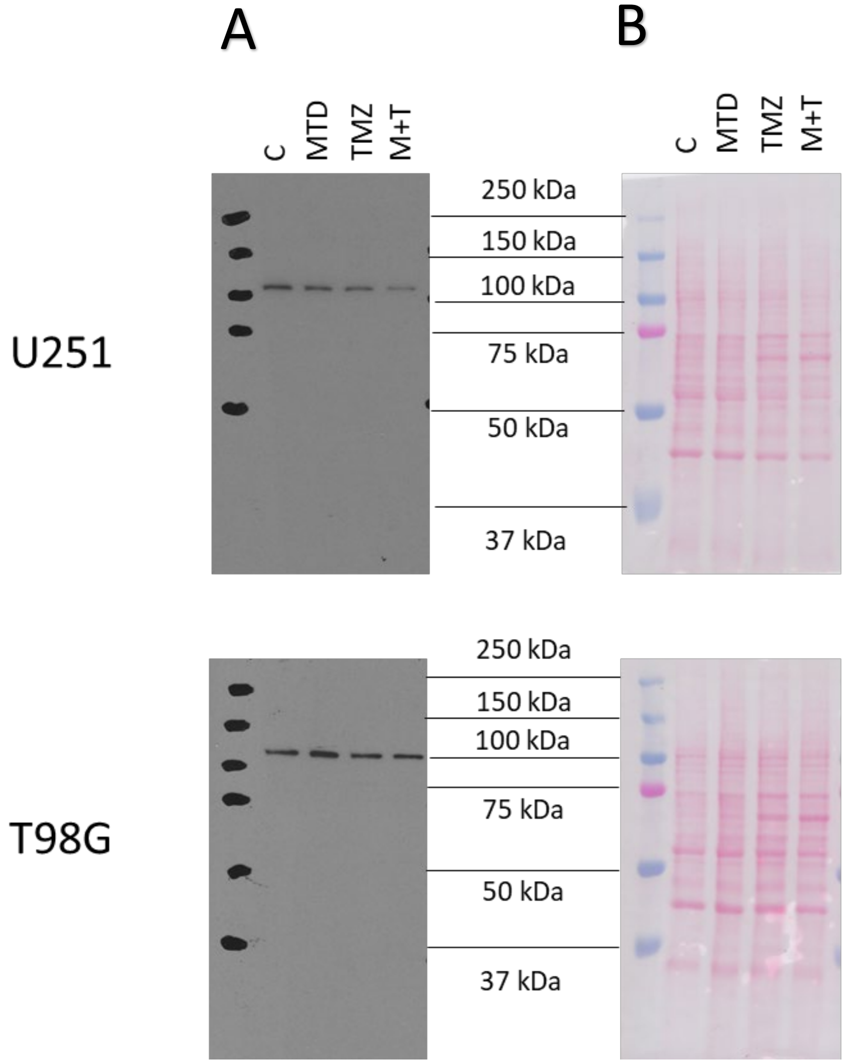

Supplementary Figure S9. Examples of original images of immunoblots and Ponceau staining. U251 and T98G glioblastma cells were treated with MTD (10 µM), TMZ (400 mM), or MTD+TMZ for 72 hours, and cell homogenates were subjected to SDS-PAGE and then, before immunoblotting with antibody to PARP1 protein, stained with Ponceau S to normalize loading. Shown are examples of Western blots of samples prepared from cells treated for 72 hours with the tested drugs and their combinations (A) and images of the corresponding nitrocellulose membranes stained with Ponceau S (B).
